# Challenges posed by COVID‐19 in cancer patients: A narrative review

**DOI:** 10.1002/cam4.4519

**Published:** 2021-12-23

**Authors:** Zeinab Mohseni Afshar, Rezvan Hosseinzadeh, Mohammad Barary, Soheil Ebrahimpour, Amirmasoud Alijanpour, Babak Sayad, Dariush Hosseinzadeh, Seyed Rouhollah Miri, Terence T. Sio, Mark J. M. Sullman, Kristin Carson‐Chahhoud, Arefeh Babazadeh

**Affiliations:** ^1^ Clinical Research Development Center Imam Reza Hospital Kermanshah University of Medical Sciences Kermanshah Iran; ^2^ Student Research Committee Babol University of Medical Sciences Babol Iran; ^3^ Students’ Scientific Research Center (SSRC) Tehran University of Medical Sciences Tehran Iran; ^4^ Infectious Diseases and Tropical Medicine Research Center Health Research Institute Babol University of Medical Sciences Babol Iran; ^5^ Faculty of Medicine Semmelweis University Üllői út 26 Budapest Hungary; ^6^ O. O. Bogomolets National Medical University Kyiv Ukraine; ^7^ Cancer Research Center Cancer Institute of Iran Tehran University of Medical Science Tehran Iran; ^8^ Department of Radiation Oncology Mayo Clinic Phoenix Arizona USA; ^9^ Department of Social Sciences University of Nicosia Nicosia Cyprus; ^10^ Department of Life and Health Sciences University of Nicosia Nicosia Cyprus; ^11^ Australian Centre for Precision Health University of South Australia Adelaide Australia

**Keywords:** cancer, COVID‐19, immunodeficiency, SARS‐CoV‐2

## Abstract

A novel coronavirus, or severe acute respiratory syndrome coronavirus 2 (SARS‐CoV‐2), was identified as the causative agent of coronavirus disease 2019 (COVID‐19). In early 2020, the World Health Organization declared COVID‐19 the sixth public health emergency of international concern. The COVID‐19 pandemic has substantially affected many groups within the general population, but particularly those with extant clinical conditions, such as having or being treated for cancer. Cancer patients are at a higher risk of developing severe COVID‐19 since the malignancy and chemotherapy may negatively affect the immune system, and their immunocompromised condition also increases the risk of infection. Substantial international efforts are currently underway to develop specific methods for diagnosing and treating COVID‐19. However, cancer patients’ risk profiles, management, and outcomes are not well understood. Thus, the main objective of this review is to discuss the relevant evidence to understand the prognosis of COVID‐19 infections in cancer patients more clearly, as well as helping to improve the clinical management of these patients.

## 1 INTRODUCTION

Since the emergence of severe acute respiratory syndrome coronavirus 2 (SARS‐CoV‐2), many infected individuals and the high mortality rate have caused a significant burden on public health worldwide.^1^ All of the risk factors which increase the severity or mortality of the current coronavirus disease 2019 (COVID‑19) have not yet been identified but are more severe in immunocompromised patients.^2^ Patients with malignant tumors are one important immunosuppressed group in the population. Cancer patients with hematologic malignancies who are receiving T cell‑depleting therapies or immunosuppressive therapy, or have had allogeneic hematopoietic cell transplantation, are at a higher risk of acquiring severe infection.^3^ Furthermore, increased hospitalization and nosocomial transmission of SARS‐CoV‐2 are another reason for a surge in infections in this group. Additionally, glucocorticoids, used in various therapy protocols, suppress both humoral and cellular immunity.

Moreover, surgery is another factor that makes cancer patients more susceptible to all kinds of infections, including viral diseases.^4^ Finally, psychological disorders caused by the COVID‐19 pandemic, such as anxiety and depression, could negatively affect adherence to chemotherapy or other treatments, making this population more vulnerable.^5^ All the above problems increase COVID‐19 severity, chances of hospitalization, the likelihood of intensive care unit (ICU) admission, need for mechanical ventilation, and mortality in this high‐risk population. The worst COVID‐19 outcomes, including acute respiratory distress syndrome, septic shock, acute myocardial ischemia, and death, would also be more likely in cancer patients undergoing surgery or chemotherapy for 14–30 days before getting infected with the virus.^6^, ^7^

## 2 IMMUNOPATHOGENESIS OF MORE SEVERE SARS‐COV‐2 INFECTION IN CANCER PATIENTS

The humoral and cellular immune systems play an essential role in defending against viral infections. Neutralizing antibodies effectively prevent viral entry, whereas cellular immunity is vital in activating CD4^+^ helper T cells, required for triggering humoral immunity, and CD8^+^ cytotoxic T cells, which are vital for the recognition and destruction of infected cells.^8^ Studies on previous coronavirus infections, such as Middle‐East Respiratory Syndrome (MERS) and SARS, have shown that CD8^+^ T cell responses are directly linked to the severity of the disease.^9^ Thus, cancer patients are at exceptionally high risk of COVID‑19 infection because of the therapies they receive, such as anti‐CD20 medications, Janus kinase inhibitors (JAKi), or Bruton tyrosine kinase inhibitors (BTKi), which weaken humoral immunity through inhibiting B cell function. These immunosuppressive agents can also cause T cell dysfunction and inhibition.^10^ Patients on active cytotoxic chemotherapy or who recently had hematopoietic stem cell transplants (HSCT) usually suffer from myelosuppression, resulting in an innate and adaptive immunodeficiency.

Furthermore, cancer development often weakens the immune system.^11^ It has also been proposed that having a history of smoking, which may include a substantial proportion of those with cancer, aggravates the situation by overexpressing immunosuppressive cytokines, suppressing the induction of pro‐inflammatory danger signals, impairing dendritic cell maturation, and enhancing immunosuppressive regulatory T lymphocyte numbers.^12^ Tobacco use also leads to a significant increase in the gene expression of angiotensin‐converting enzyme 2 (ACE2), the binding receptor for SARS‐CoV‐2, further elevating susceptibility to COVID‐19 infection.^13^

## 3 RISK FACTORS INFLUENCING INFECTION SEVERITY IN ONCOLOGY PATIENTS

Recent studies have shown that cancer patients with COVID‐19 are more likely to be admitted to the ICU, require mechanical ventilation, or die.^14^ In addition, a delayed admission time, due to the similarity between COVID‐19 and cancer symptoms, might be another reason for the more likely progression to severe disease. In one study, the case fatality rate reached 5.6% among cancer patients, while the COVID‐related mortality in the general population has been reported to be 2.3%.^15^ Therefore, the risk factors that may worsen the outcomes among cancer patients should be carefully examined. In a study investigating risk factors for developing severe complications in cancer patients, among those receiving antitumor treatment within 14 days of a COVID‐19 diagnosis, undergoing chemotherapy, radiotherapy, targeted therapy, immunotherapy, and the presence of patchy consolidation in the first computed tomography (CT) scan of the lungs on admission were identified as significant risk factors.^16^ Moreover, due to the more potent myelosuppressive therapy they received, patients with hematologic malignancies are more likely to develop a severe infection than those with solid tumors.^17^

As components of a chemotherapy regimen, treatment with high‐dose corticosteroids and immune checkpoint inhibitors (ICIs) have been independently associated with SARS‐CoV‐2 infection‐related severity and mortality.^18^ Treatment regimens containing JAKi or BTKi may also put these patients at a higher risk of developing severe infections.^19^ Another vital point to consider is that symptoms and radiological features of ICI‐induced pneumonitis can be overlapping with those of COVID‐19‐related pneumonia. For the latter, dexamethasone and remdesivir have shown encouraging results.^20^ On the other hand, the mainstay of treatment in ICI‐induced pneumonitis is immunosuppressive therapy. It has been speculated that immunosuppression may be associated with an increased risk of progression to severe COVID‐19, especially during the early stage of infection.^20^ Therefore, although the distinction between these two entities could be challenging for clinicians, it is highly warranted.

**FIGURE 1:**
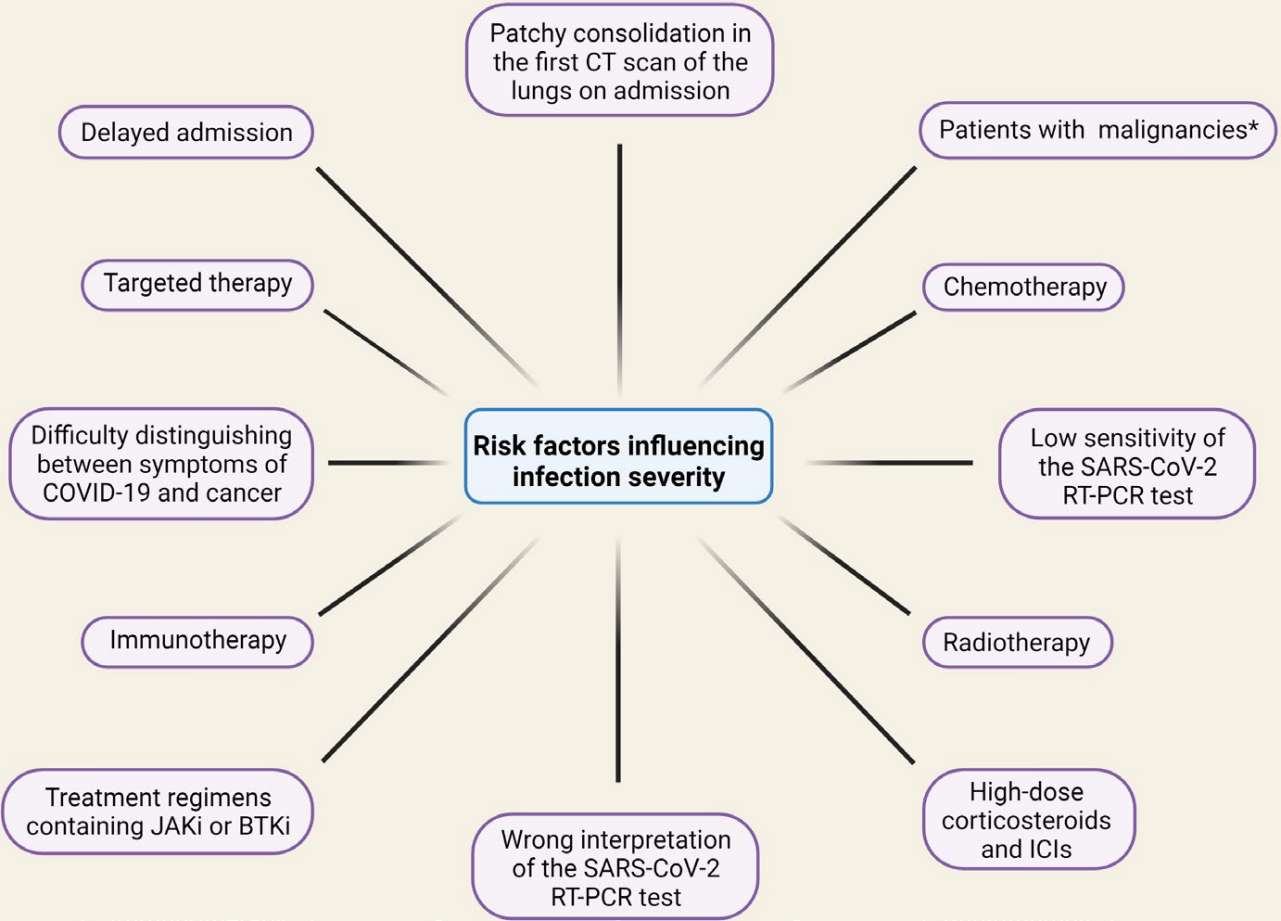
The risk factors influencing infection severity in cancer patients. Several factors can increase the risk of COVID‐19 in cancer patients. These risk factors are delayed admission, low sensitivity, or wrong interpretation of the SARS‐CoV‐2 RT‐PCR tests. In some cases, the initial diagnosis may not be correct due to the similarity of cancer symptoms and COVID‐19. There are also risk factors for cancer treatment, including chemotherapy, targeted therapy, radiotherapy, immunotherapy, and treatment regimens containing JAKi or BTKi. Treatment with high‐dose corticosteroids and ICIs can also increase the chance of infection. Observing patchy consolidation in the first CT scan of the lungs on admission is also a risk factor for increasing the severity of infection. Also, patients with some cancers are more susceptible to infection than others. Abbreviations: BTKi, Bruton tyrosine kinase inhibitors; CT, computed tomography; ICIs, Immune checkpoint inhibitors; JAKi, Janus kinase inhibitors. ^*^Gastrointestinal, thoracic (particularly non‐small cell lung carcinoma), and head and neck cancers

On the other hand, the sensitivity of the SARS‐CoV‐2 RT‐PCR test is quite low, and the consequences of wrong interpretation can be too severe, to fully trust a negative result in clinically suspicious cases. Thus, additional parameters must be considered when assessing patients treated with ICIs with suspicion of COVID‐19.^20^ Based on imaging modalities, laboratory assays, SARS‐CoV‐2 RT‐PCR test, and if necessary, bronchoscopy with bronchoalveolar lavage, a diagnostic blueprint can be proposed to aid clinicians in daily practice to establish the right diagnosis between these two similar diseases, leading to the initiation of the correct course of treatment.^20^ Moreover, studies conducted among patients with various malignancies have demonstrated that the three most common malignancies in COVID‐19‐infected patients were gastrointestinal, thoracic (particularly non‐small‐cell lung carcinoma), and head and neck cancers.^21^ With this in mind, patients with lung neoplasms should be one of the top priority groups for COVID‐19 prevention programs, such as vaccination. Therefore, during the COVID‐19 pandemic, these patients should be actively screened for fever and respiratory symptoms and be kept separately from suspected cases of COVID‐19^22^ (Figure 1).

## 4 CHALLENGES OF ONCOLOGISTS DURING THE COVID‐19 PANDEMIC

The epidemic spread of this novel coronavirus has imposed significant challenges on the clinical practice of oncologists, especially for diagnosis and therapy. Studies have shown that the rate of cancer diagnosis and newly detected malignancies were significantly lower during the pandemic than for the same period before this outbreak.^23^ A systematic review of 62 studies measured and reported at least one delay or disruption in cancer health care because of the COVID‐19 pandemic.^24^ The studies addressed 38 different categories of delays and disruptions with established or potential impact on the treatment plan, diagnosis, or health‐service process. Most of the delays and disruptions in this study were as follows: reduction in any routine activity of cancer services, including the visits; reduction in the number of cancer surgeries; delay in radiotherapy; and delay, reschedule, or cancellation of outpatient visits. According to this survey, up to 77.5% of the patients reported interruption in any stage of treatment.^24^ Another interesting study by Ghahramani‐Asl et al^25^ indicated the possible beneficiary effect of partial lung irradiation in COVID‐19 patients. In this study, the authors imported the CT images of 10 COVID‐19 patients into a specific treatment planning .system to anatomically define and contour the volumes of the pulmonary lesions, the lungs, and other nearby organs.^25^ For the first time, they report the feasibility and acceptability of using this treatment planning system in the volumetric assessment of COVID‐19 lung lesions and its validity in determining the location of pulmonary lesions as a target for 3D conformal radiation therapy. Thus, if proven safe and effective in future studies, this modality could also be considered in COVID‐19 patients’ treatment plans.^25^

Oncologists must carefully determine the risk of COVID‐19 exposure in their patients. Since a diagnosis of cancer places infected patients at significantly increased risk of morbidity, including the need for mechanical ventilation, or mortality, it would be appropriate to decrease unnecessary exposure to COVID‐19 for cancer patients in the health care system. However, the consequences of delayed diagnosis or treatment in common cancers must also be carefully considered,^26^, ^27^ and the decision about whether to continue maintenance therapy should be made individually. Some hematologic cancers, such as acute leukemia, and many solid tumors, including lung or pancreatic cancers, require urgent diagnosis and therapy. In contrast, other common early‐stage neoplasms (e.g., breast, prostate, cervical, or non‐melanoma skin cancers) do not need immediate intervention.^28^ For example, maintenance rituximab in follicular and mantle cell lymphomas are clear examples of where changes to maintenance therapy are necessary, as this anti‐CD20 agent could significantly inhibit B cells, resulting in a much lower immune response to pathogens like SARS‐CoV‐2. Nevertheless, a delay in treating metastatic cancers can result in a much worse prognosis, significantly higher disease progression, and more hospitalizations. However, it is worth mentioning that some early‐stage hormone‐positive breast cancer patients can be kept on their hormone therapy if needed.^29^

A study on the effect of the current pandemic on cancer patients in Iran argued that cancer patients in developing countries with limited resources encounter more serious problems during outbreaks.^30^ This is mainly because the healthcare systems do not prioritize these patients. In addition, the lack of appropriate guidelines for their condition worsens the situation. Other problems in these countries include using radiation treatment centers as COVID‐19 referral centers, lack of available hospital beds because they are allocated to cancer care, and deployment of the same nursing and hospital staff in cancer treatment centers COVID‐19 wards.^30^ Moreover, timely delivery of radiotherapy is essential for patients, and any interruptions in radiation therapy may lead to cancer recurrence. Thus, another important issue is the irregular visits of patients for their treatments due to the fear of getting infected.^30^

At the pandemic’s beginning, the overall desire was to postpone nonurgent chemotherapy interventions in cancer patients. However, it is currently believed that routine antineoplastic therapy should not be delayed or stopped in patients without suspected or confirmed SARS‐CoV‐2.^31^ Conversely, suppose a SARS‐CoV‐2 infection is suspected. In that case, the patient should be quarantined. The antineoplastic therapy should be delayed for up to 14 days,^31^ but if the infection is confirmed, delaying or discontinuing chemotherapy is strongly recommended, as it significantly increase the risk of morbidity and mortality.^4^

Moreover, surgery or radiotherapy in cancer patients is strongly discouraged in the acute phase of SARS‐CoV‐2 infection.^32^, ^33^ Moreover, although more studies are required, another concerning issue is the interactions between anti‐COVID‐19 therapies (e.g., antiviral agents and monoclonal antibodies) with antineoplastic regimens, such as chemotherapy, hormonotherapy, targeted therapy, and immunotherapy.^34^ Another critical issue to be considered is the similarity between some symptoms of COVID‐19 and cancer at the time of diagnosis (e.g., fever or cough), which may result in a misdiagnosis or delayed diagnosis of some malignancies, such as acute leukemia, primary mediastinal lymphoma, or lung cancer.^35^ In addition, the predominant peripheral ground‐glass opacities (GGOs) or predominant lung consolidations of the lower lobes are a common radiographic presentation of metastatic lung cancers, differentiating a new COVID‐19 infection from the so‐called neoplasms would be challenging. In these cases, positron‐emission tomography/CT scans would be appropriate diagnostic options for differentiating active lesions from new infections imposed upon the underlying malignant lesions.^36^ Comorbidities are another vital determinant of morbidity in cancer patients. A previous study showed that mortality was significantly higher in SARS‐CoV‐2‐infected cancer patients, although the comorbidities, especially diabetes mellitus, were more prevalent in nonmalignant patients.^37^ Moreover, the probability of a positive SARS‐CoV‐2 RT‐PCR test was significantly higher in nonmalignant patients. Regression analysis showed that the risk of death in COVID‐19 cancer patients was about nine times greater than in other patients. Also, the patients who needed mechanical ventilation had a significantly higher mortality rate.^37^

Low anti‐SARS‐CoV‐2 IgG antibody titers are another important risk factor in cancer patients, making them more vulnerable to the infection. Although numerous studies have already discussed this phenomenon,^38^, ^39^, ^40^ little is known about the pathophysiology of this condition.^41^ Moreover, there was a significant difference in SARS‐CoV‐2 IgG seroconversion among cancer patients undergoing various treatment plans. For instance, Thakkar et al.^42^ showed that cancer patients with hematological malignancies who received anti‐CD20 antibody regimens and undergone stem cell transplantation had significantly lower seroconversion than other cancer patients. Furthermore, interestingly, their findings concluded that cancer patients who received immunotherapy, including anti‐PD‐1/PD‐L1 monoclonal antibodies, developed 100% seroconversion for SARS‐CoV‐2. Thus, these lower seroconversion rates in cancer patients necessitate the importance of rigorous clinical monitoring and vaccination strategies in these susceptible populations.^42^ Nevertheless, even though such decreased anti‐SARS‐CoV‐2 antibodies were not detected in asymptomatic COVID‐19 cancer patients, more clinical studies are mandated to better understand this difference in asymptomatic patients since they play an essential role in the COVID‐19 transmission chain.^43^

**FIGURE 2:**
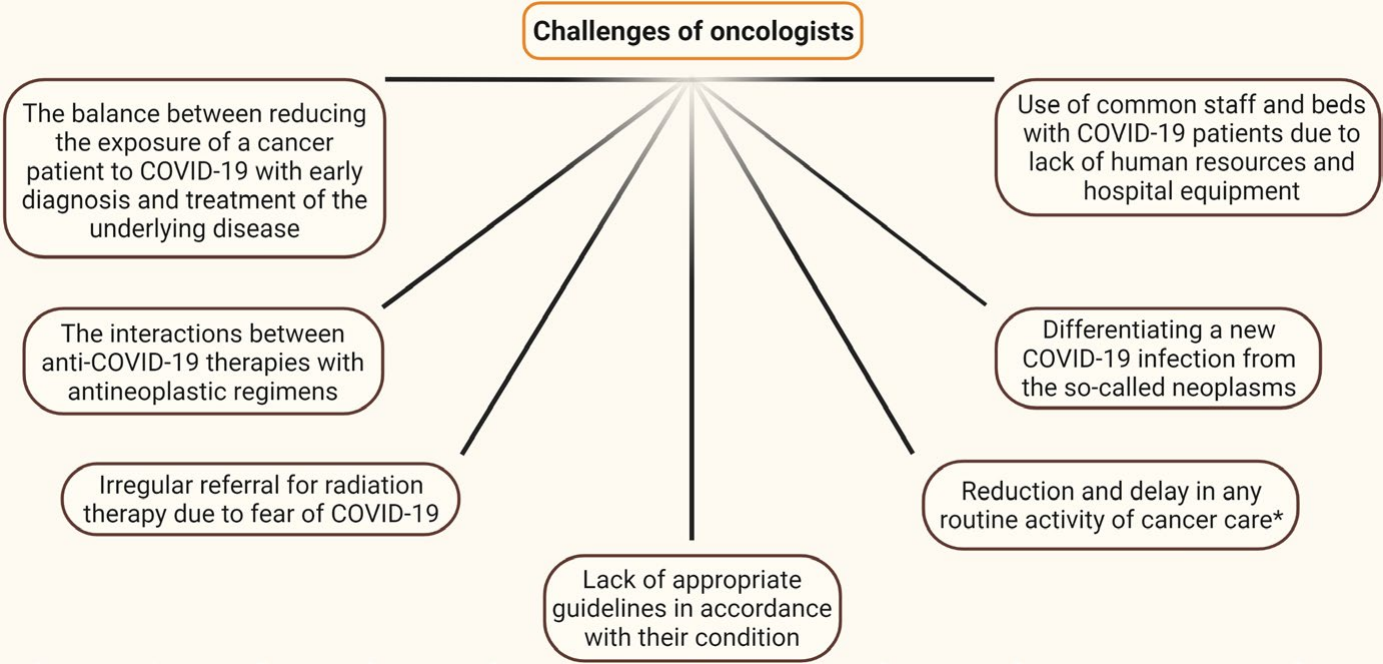
Challenges of oncologists in the face of COVID‐19. Due to the COVID‐19 pandemic, oncologists face many challenges in treating cancer patients. One of these challenges is using shared staff and beds for cancer patients with COVID‐19 due to a lack of human resources and hospital equipment. Other challenges include the fear of developing COVID‐19. Many patients delay seeing a doctor because of this fear, which delays diagnosis, treatment, or even radiation therapy. Lack of appropriate guidelines for their condition also worsens the condition of patients with cancer. Interactions between antineoplastic regimens and anti‐SARS‐CoV‐2 treatment are a major challenge for physicians. One of the most important challenges was accurately detecting COVID‐19 from the so‐called neoplasms. Reducing or delaying any routine cancer care activity is also a challenge. ^*^Including the visits, reduction in the number of cancer surgeries, delay in radiotherapy, and delay, reschedule, or cancellation of outpatient visits

In summary, the unprecedented burden of COVID‐19 on healthcare systems worldwide has a significant impact on cancer care. First, despite limited data, cancer patients seem to be more susceptible to the more catastrophic outcomes from the infection, including increased need for mechanical ventilation^6^ and mortality rates^37^, ^44^, ^45^, ^46^, ^47^ (Table 1). Second, the diagnosis might be withheld as screening programs and diagnostic services have been decreased or suspended in many countries, and patients, wary of exposing themselves to the risk of infection, have been more reluctant to present to healthcare services.^48^ Third, Treatment routes have been modified to minimize potential exposure of cancer patients to SARS‐CoV‐2 and to reduce the risk during surgery or radiation therapy. Fourth, certain aspects of ongoing care have been deprioritized to enable health systems to respond to the current pandemic, resulting in suboptimal or delayed care for cancer patients. Fifth, many clinical trials have been suspended, reducing current therapy options for their participants, and jeopardizing longer‐term therapy development ^48^ (Figure 2).

**TABLE 1:** The outcome of cancer patients in different COVID‐19 studies

First author, year	Location	Type of malignancy included	Duration of study, weeks	Total number of patients with malignancy	Total number of hospitalized patients with malignancy	Median age of patients with malignancy, year	Deceased patients, *N* (%)	Deceased patients with malignancy, *N* (%)
Cook, 2020^49^	UK and Italy	Myeloma	15	75	72	73	30 (40)	41 (55)
Ferrara, 2020^50^	UK and Italy	AML	4	10	10	60	5 (50)	5 (50)
Mato, 2020^51^	Multiple countries	CLL	11	198	178	71	73 (37)	66 (33)
Yigenoglu, 2020^52^	Turkey	Hematological malignancies	15	740	452	56	343 (46)	102 (14)
Song, 2020^53^	China	Multiple cancers	12	248	101	63	38 (15)	2 (0.8)
Chai, 2020^54^	China	Multiple cancers	52	166	166	65	60 (36)	49 (29)
Mousavi, 2020^55^	Iran	Multiple cancers	8	33	33	64	16 (48)	13 (39)
Aboueshia, 2020^56^	USA	Multiple cancers	8	57	57	59	40 (70)	7 (12)
Condom, 2020^57^	Spain	Multiple cancers	12	24	24	69	11 (45)	11 (45)
Biernat, 2020^58^	Poland	Hematological malignancies	4	10	10	58	8 (80)	7 (70)
Aries, 2020^59^	Netherlands	Hematological malignancies	8	35	24	69	12 (34)	14 (40)
Booth, 2020^60^	UK	Hematological malignancies	8	66	66	73	25 (38)	34 (52)
Engelhardt, 2020^61^	Germany	Multiple myeloma	12	21	17	59	4 (19)	0 (0)
Fox, 2020^16^	UK	Hematological malignancies	4	54	51	63	18 (33)	19 (35)
He, 2020^62^	China	Hematological malignancies	3	13	13	35	6 (46)	8 (62)
Hultcrantz, 2020^63^	USA	Myeloma	7	100	74	68	42 (42)	18 (18)
Infante, 2020^64^	China	Hematological malignancies	4	41	29	76	19 (47)	15 (37)
Lattenist, 2020^65^	Belgium	Hematological malignancies	8	12	12	74	3 (25)	6 (50)
Malard, 2020^66^	Multiple countries	Hematological malignancies	4	25	25	72	8 (32)	10 (40)
Martin‐Moro, 2020^67^	Spain	Hematological malignancies	5	34	34	73	15 (44)	11 (32)
Passamonti , 2020^68^	Italy	Hematological malignancies	12	536	451	68	196 (37)	198 (37)
Razanamahery, 2020^69^	France	Hematological malignancies	8	20	20	69	7 (35)	6 (30)
Sanchez‐Pina, 2020^70^	Spain	Hematological malignancies	4	39	34	65	16 (41)	14 (40)
Scarfo, 2020^71^	Multiple countries	CLL	10	190	169	72	64 (34)	56 (29)
Shah, 2020^72^	UK	Hematological malignancies	8	80	80	73	28 (35)	28 (35)
Wang, 2020^73^	USA	Myeloma	8	58	36	67	28 (48)	14 (24)

Abbreviations: AML, Acute myeloid leukemia; CLL, Chronic lymphocytic leukemia.

## 5 CHALLENGES OF CANCER SURGERY DURING THE PANDEMIC

During this outbreak, specific surgical recommendations have been made for common malignancies in cancer‐specific guidelines. For instance, for gynecological cancers, surgeries are recommended to be postponed, with only emergent or urgent surgeries to be performed. Radiotherapy and concomitant chemoradiotherapy could be used instead, particularly for digestive neoplasms, laparoscopic surgery could also be undertaken with strict precautions. Furthermore, palliative therapy, such as stenting for esophageal cancers, can also be considered. However, it should be noted that delayed oncologic surgery may lead to cancer progression, resulting in the tumor no longer being resectable, with the associated worse survival outcomes.^74^, ^75^ Another study provided initial estimates and reference points for future research on the impact of the COVID‐19 pandemic on oncological resection rates.^76^ As expected, the participating surgical departments perceive a reduction in tumor resections of all types. On average, the number of resections (for all questioned cancer types) was reduced by almost a third, consistent with another publication^77^ that estimates 38% of all cancer surgeries in all disciplines worldwide were canceled due to the COVID‐19 crisis.^76^ Thus, it is recommended that patients who need to be operated on should at least have an adverse reverse transcriptase‐polymerase chain reaction (RT‐PCR) for SARS‐CoV‐2.^3^ Also, in this situation, high‐risk aerosolizing procedures should be avoided, appropriate personal protective equipment (PPE), such as N95 masks, goggles, gowns, and gloves, should be done by all health care workers, and such procedures should be performed in unfavorable pressure rooms, where possible.^19^

## 6 CHALLENGES FOR CHEMOTHERAPY DURING THE PANDEMIC

Chemotherapeutic agents predispose patients to infections through impairing bone marrow function, leading to thrombocytopenia and neutropenia. The risk of infection is highest when their absolute neutrophil count (ANC) is the lowest, usually 7–12 days after each chemotherapy session.^19^ Some cytotoxic agents (e.g., temozolomide, cyclophosphamide, paclitaxel, cisplatin, methotrexate, and fludarabine) may induce severe damage to the bone marrow and alemtuzumab, leading to lymphopenia and an increased risk of infection.^78^ Interactions between antineoplastic agents and potential SARS‐CoV‐2 infection therapies should also be considered. For example, some chemotherapeutic agents, such as vinca alkaloids (vincristine and vinblastine) and taxanes (docetaxel and paclitaxel), show significant interactions with protease inhibitors (e.g., atazanavir, lopinavir, and ritonavir), which were commonly used for treating SARS‐CoV‐2‐infected patients at the beginning of the pandemic. Moreover, many other agents, such as tyrosine kinase inhibitors (dasatinib and ibrutinib), may interact with heparin, a commonly used anticoagulant in hospitalized patients. In addition, rituximab, a monoclonal anti‐CD20 antibody, has significant interactions with tocilizumab, an approved interleukin‐6 (IL‐6) antagonist commonly used in severe COVID‐19 patients.^79^

A potential solution to the increased risk of infection and increased severity in patients undergoing chemotherapy could be through the use of low‐dose metronomic chemotherapy with different agents and schedules. This intervention can hopefully control the tumors and has more favorable safety profiles. In addition to the approaches mentioned above, the continuation of cancer care during the pandemic would be enhanced if oral administration of the medication was possible.^80^ Nevertheless, SARS‐CoV‐2 RT‐PCR testing should be carried out before initiating the treatment in all cases with an urgent need for chemotherapy.

## 7 CHALLENGES OF RADIOTHERAPY DURING THE PANDEMIC

Radiation therapy is one of the main treatment options for malignancies. This intervention can lead to immunosuppression by inducing bone marrow suppression and lymphopenia. Therefore, radiotherapy can put the patient at increased risk of infection, morbidity, or mortality.^81^ Patients undergoing this type of treatment would usually continue their treatment for several weeks. However, as most staff at radiotherapy centers had been off work since the beginning of the pandemic, the intervals between radiotherapy sessions have increased, possibly leading to a decrease in their therapeutic efficacy. Thus, if treatments were postponed, like elective surgeries, adverse clinical outcomes may become inevitable, as this modality usually consists of multi‐fraction courses of therapy that require daily visits to the clinic.^82^ Moreover, some of the typical and nonspecific side effects of radiotherapy, and even some cancer manifestations (e.g., low‐grade fever, cough, sore throat, and rhinorrhea), mimic COVID‐19 symptoms, differentiating between the two can be challenging.^83^

Several protocols have been implemented in different centers to reduce the burden of this epidemic. Many centers provide radiotherapy for patients with negative COVID‐19 screening results, while others recommend asymptomatic patients to wait until a sufficient isolation period has passed following close contact with a suspected or confirmed patient. For confirmed COVID‐19 patients who have fully recovered, almost all centers recommend starting radiotherapy after being quarantined for at least 14 days. However, it should also be noted that unnecessarily delaying these sessions would adversely affect cancer management.^84^ Furthermore, almost all radiotherapy centers have planned areas for previously infected COVID‐19 patients to undergo treatment, separated from noninfected patients. Patients should also be instructed to keep the interpersonal spacing of at least 2 m in the general waiting areas. Disinfecting the treatment bed and surrounding accessories during the treatments would also be helpful. Some centers may classify cancer patients into confirmed and suspected cases of COVID‐19, cases that should be medically isolated, and cases with negative screening results. In the early stages of malignancy, negatively screened patients should only receive radiation therapy if deemed absolutely necessary. At the same time, those with locally advanced tumors are recommended a neoadjuvant chemotherapy regimen or hormone therapy first and then to continue with radiotherapy after some delay.^85^ Patients already receiving radiotherapy should be individually assessed about whether to continue therapy with the previous dose or reduce the dose’s intensity.^86^ Moreover, using proton beam therapy, stereotactic body radiation, or a hypo‐fractionated schedule can also be considered to decrease the risk of radiation‐related immunosuppression.^78^

High‐risk patients who are mandated to undergo radiotherapy should be treated as the last case of the day, with all personnel wearing appropriate PPE, including N95 respirators, surgical masks, and gloves, depending on medical policies, available supplies, and procedural risks. All patients and accompanying individuals must also be required to wear surgical masks. Some centers even implement mandatory twice daily monitoring of temperature for all staff. Educational information about personal hygiene, the importance of handwashing, and the appropriate methods of wearing masks should be highly prioritized in the patient care programs. Moreover, interventional radiology staff should follow the standard precautions, primarily including personal and hand hygiene, proper ward ventilation, and disinfection of instruments, to minimize the risk of nosocomial infections.^82^, ^87^

## 8 CHALLENGES OF IMMUNOTHERAPY DURING THE PANDEMIC

Immunotherapy is another therapeutic modality for treating‐specific cancer types. This therapeutic option includes vaccines, ICIs, T cell transfer therapy, and immunomodulators. Despite being beneficial in treating malignancies, these agents have side effects like hyperactivated T cell responses, directly affecting, and harming normal tissues. Hence, the decision to initiate or continue immunotherapy during this outbreak or during the acute phase of SARS‐CoV‐2 infection should be made individually. These agents’ most significant adverse effects include thrombocytopenia, prolonged lymphopenia, pneumonitis, cytokine release syndrome (CRS), and increased vascular permeability, leading to pleural effusion, or pulmonary edema.^88^ More recently, targeted therapies, such as selective Fms‐related receptor tyrosine kinase 3 (FLT3, also known as CD135) inhibitors (e.g., midostaurin, quizartinib, crenolanib, and gilteritinib), BCL‐2 inhibitors (e.g., venetoclax), or isocitrate dehydrogenase (IDH) inhibitors (e.g., ivosidenib and enasidenib), have been used for some neoplasms (e.g., acute myeloid leukemia or acute lymphocytic leukemia). The risk of severe respiratory failure in patients treated with these agents, who are concurrently infected with SARS‐CoV‐2, has been raised and should be validated in future studies.^89^.

## 9 CHALLENGES OF BONE MARROW TRANSPLANTATION DURING THE PANDEMIC

Patients who are candidates for bone marrow transplantation are better to defer their surgery due to the subsequent need for long‐term immunosuppression. This treatment modality weakens the immune system, which predisposes the patient to an increased risk of infection for 3 months after the transplant, although complete recovery may take up to a year in some cases.^90^ HSCT (hematopoietic stem cell transplantation) has been dramatically affected by the COVID‐19 pandemic in some ways since donors and recipients must both test negative for COVID‐19 for the procedure to be feasible and successful. If potential donors are infected with SARS‐CoV‐2, the donation should be delayed until a full recovery has been made. Thus, having a backup donor might help in this situation.^91^

## 10 PREVENTIVE MEASURES AGAINST SARS‐COV‐2 INFECTION IN CANCER PATIENTS

The first question would be whether protective measures needed for cancer patients are any different from those needed for immunocompetent individuals. It should be emphasized that standard personal protection, similar to healthy individuals, should be worn by cancer patients on active therapy and those who are cancer‐free.^92^ However, more vigilant and intensive provisions or treatment plans should be considered for SARS‑CoV‑2‐infected cancer patients, especially the elderly or those with other comorbidities. In addition, SARS‑CoV‑2‐induced pneumonia rapidly spreads through person‐to‐person transmission by droplets, and because cancer patients should usually be hospitalized for their therapy and disease surveillance, they are at higher risk of SARS‐CoV‐2 exposure. Therefore, the most sensible strategy for these patients in this outbreak would be to suspend adjuvant chemotherapy or elective surgery for stable patients to decrease hospitalization and the need for multiple hospital visits and, subsequently, close contacts COVID‐19 suspected patients or healthcare workers.^93^

Nevertheless, if cancer therapy must be undertaken, self‐isolation following treatment may enable patients to delay or avoid being infected with COVID‐19, which is particularly important following chemotherapy. It would also be helpful if outpatient clinics used telehealth options, such as telephone‐ or video‐conferencing appointments for their patients.^94^ However, if attending the clinics cannot be avoided, patients should be asked to wait outside until their turn to avoid crowding in one area and reduce their exposure to other patients and healthcare personnel. Precautions could also include screening patients and visitors for COVID‐19 upon arrival.^95^

Oral chemotherapy may be another good way of avoiding unnecessary hospital admissions.^95^ For patients who require urgent malignancy treatment, proper isolation measures should be considered, such as reducing chemotherapy intensity, decreasing the frequency of cancer care sessions, or establishing off‐site cancer care facilities.^96^ The most important and effective strategy to prevent COVID‐19 is “social distancing,” the primary intervention to reduce the spread of this infection. This strategy is significantly disrupted by any engagement of cancer patients with the health care settings, including clinic visits, surgical stays, infusion sessions, radiation planning, treatment appointments, hospital admissions, phlebotomies for laboratory tests, and radiographic studies, all of which provide potential opportunities for viral transmission.^97^ In addition to receiving COVID‐19 vaccines, these patients should take other precautions to reduce their risk of infection.^98^ Since secondary bacterial infections may superimpose on viral infections, vaccination against *Streptococcus pneumoniae* should be recommended for this at‐risk population.^99^

## 11 COVID‐19 AND CANCER IN CHILDREN

Although severe COVID‐19 infection is rarely believed in children, some studies have shown a higher illness severity among immunocompromised infants and younger children. Childhood cancers pose many challenges during the current COVID‐19 pandemic. Since most childhood malignancies are aggressive and need urgent treatment, delaying treatment might not be appropriate for these patients.^100^ Therefore, strategies should be undertaken to prevent and decrease the risk of exposure to SARS‐CoV‐2 in children receiving intensive chemotherapy or stem cell transplants, with isolation being the best option. Families are also advised to strictly adhere to standard preventive precautions, such as social distancing.^101^

## 12 ANTITUMOR MEDICATIONS THAT CAN BE POTENTIALLY USED FOR COVID‐19 TREATMENT

A pro‐inflammatory state resulting from a cytokine storm is believed to deteriorate significantly COVID‐19‐ patients’ condition. Hence, it is proposed that a group of immunosuppressive therapies may have a protective role in helping infected patients by reducing the intensity of the cytokine storm and thereby preventing further lung tissue damage.^102^ Several medications used for chemotherapy or immunotherapy in cancer patients may also effectively inhibit COVID‐19 by stimulating the immune response.^103^ Important examples are certain TKIs, which have proven effective in treating SARS, MERS, and COVID‑19 infections. However, TKIs, such as erlotinib, an FDA‐approved inhibitor of the epidermal growth factor receptor, which is used to treat non‐small cell lung (NSCLC) and pancreatic cancers, may have interactions with antiretroviral agents, such as lopinavir, and ritonavir, which were used to treat COVID‐19 early in the pandemic.

Nonetheless, these agents can themselves be good options for managing SARS‐CoV‐2. Moreover, JAK inhibitors (e.g., ruxolitinib, baricitinib, and tofacitinib) have also shown promise in managing COVID‐19 through hyper‐reactivating the immune response to the infection.^104^ However, the additive risk of thrombotic events caused by a SARS‐CoV‐2 infection and the use of JAK inhibitors should be carefully considered.^105^

Interleukin inhibitors, which target IL‐6 and other cytokines (e.g., tocilizumab and sarilumab), are effective in specific neoplasia, including lymphoproliferative disorders, Castleman’s syndrome, and smoldering multiple myeloma,^106^ are currently being successfully utilized for suppressing the CRS during the SARS‐CoV‐2 infection.^107^ In addition, being a cytokine mediator that is included in the treatment regimens of certain cancers, such as chronic myelogenous leukemia (CML), hairy cell leukemia, melanoma, and Kaposi sarcoma,^108^ interferons can reduce viral infections and improve viral clearance.^109^ ICIs (e.g., pembrolizumab), which have revolutionized the management of a variety of solid tumors and hematological malignancies,^110^ have also been evaluated to be effective therapeutic agents for SARS‐CoV‐2 infection through decreasing viral load, and increasing antiviral‐specific function in both the CD4^+^ and CD8^+^ T cells, leading to clinical improvement, viral clearance, and attenuating lung injury.^111^ Furthermore, CCR5 inhibitors (e.g., leronlimab, thalidomide, and lenalidomide), well‐known FDA‐approved therapeutics for certain malignancies, have previously shown efficacy against SARS‐CoV‐2 infection.^112^, ^113^

## 13 CONSIDERATIONS FOR COVID‐19 VACCINES IN CANCER PATIENTS

Patients with cancer are at increased risk of adverse outcomes from COVID‐19 infections, and therefore should be prioritized for vaccination.^114^, ^115^ Currently, no COVID‐19 vaccine platform is preferred over others in cancer patients. However, it is expected that the vaccine‐induced immune response in cancer patients, particularly those undergoing immunosuppressive therapy, would be less favorable than among the immuno normal population.^116^ However, except during the intensive phase of chemotherapy, vaccine antibody responses are believed to be sufficient enough to recommend vaccination for these patients.^117^ For patients scheduled for cytotoxic chemotherapy, it is better to administer the first dose of the vaccine at least 2 weeks before chemotherapy. Nevertheless, the first dose of the vaccine can also be administered during the interval between chemotherapy sessions.^117^ Moreover, the COVID‐19 vaccination seems safe and efficient in radiation therapy patients.^118^ Since it was previously deduced that both inactivated (e.g., Sinopharm)^119^ and mRNA (e.g., Pfizer‐BioNTech)^120^, ^121^ COVID‐19 vaccines are effective in cancer patients, currently, there is no preferred vaccine for these patients. So these individuals can receive any approved vaccine under their physician’s supervision.^115^ Furthermore, it should also be noted that vaccine efficacies are shown to be lower in hematological malignancies than solid tumors.^119^

## 14 MODIFIABLE RISK FACTORS AND SUSCEPTIBILITY TO COVID‐19

Several modifiable risk factors, such as tobacco smoking, obesity, hypertension, and type 2 diabetes,^122^ may be present in cancer patients, which can increase susceptibility to COVID‐19 infection and the severity of the disease. Despite the known benefits of smoking cessation, even following a cancer diagnosis,^123^ a large proportion of people will continue to smoke.^124^ Indeed, one 2017 cross‐sectional study with over 26,000 individuals from the United States identified that people diagnosed with smoking‐related cancers were more likely to continue smoking post‐diagnosis than those diagnosed with nonsmoking‐related cancers.^125^ This is a particular concern given that ACE2 receptors are the binding site for the SARS‐CoV‐2 virus, as mentioned above, and this receptor is upregulated among current smokers.^126^ Moreover, it has been found that patients with any history of smoking are vulnerable to COVID‐19 infection, and are more likely to have a severe case resulting in ICU admission, need for mechanical ventilation, and increased mortality.^127^

Although some controversy exists whereby cigarette smoking has been associated with a lower population prevalence of COVID‐19,^128^ other studies counter these findings suggesting a slight increase in diagnosis.^129^ There is undoubtedly potential for nicotine to be considered as a therapeutic modality. However, more research is required.^130^ Nevertheless, the harms of cigarette smoking far outweigh any potential therapeutic benefits associated with continued smoking for active smokers. Therefore, encouraging smoking cessation even following cancer diagnosis will benefit cancer treatment and progression and reduce the risk of COVID‐19 infection and severity.

Similarly, a prospective study of 92 patients from a hospital in Italy evaluated the severity of COVID‐19 and obesity classes according to body mass index (BMI), identifying an increased need for mechanical ventilation and access to intensive or semi‐ICUs compared to individuals classified as having normal BMIs.^131^ Meanwhile, a study of 103 consecutive patients from the United States identified that severe obesity (BMI ≥35 kg/m^2^) was associated with more ICU admissions and invasive mechanical ventilation.^132^ A 2021 systematic review and meta‐analysis compiling evidence across nine studies confirm these findings, with severe COVID‐19 patients more likely to have a higher BMI than non‐severe patients. Patients with obesity were more likely to be severely affected by the condition and have worse disease progression.^133^ Although ACE2 expression is higher in adipose tissue than lung tissue, no current evidence suggests that COVID‐19 binds directly to adipose tissue.^132^ It is believed that pro‐inflammatory cytokines and adipokines, synthesized by adipose tissue, can weaken the immune response and thus contribute to this observed link between COVID‐19 and obesity.^134^ It is well established that obesity causes changes in the physiological function of adipose tissue, also leading to insulin resistance and chronic inflammation, and these mechanisms are known to be linked to carcinogenesis and cancer progression.^135^ Hence, encouraging weight loss among people with obesity may be beneficial in reducing the severity of SARS‐CoV‐2 infections.

## 15 THE ROLE OF TELEMEDICINE IN THE MANAGEMENT OF PATIENTS WITH CANCER

Compared with other infectious diseases, the use of telemedicine for COVID‐19 has become globally urgent in the 21st century.^136^ In China and other countries, with increase in the mortality rate and due to the quarantine situation, WHO is broadly using telemedicine to prevent the spread of the infection among individuals and continue delivering healthcare services.^137^ Exploring opportunities for the combination of telemedicine with precision medicine during the COVID‐19 pandemic includes improving welfare for cancer patients under medical treatment, resulting in the expansion of the decision process between patients and providers of healthcare programs.^138^ Interdisciplinary clinical programs utilizing telemedicine, bioinformatics, and genomics to merge these fields have also been developed for global collaboration and fighting against this unknown virus.^138^

## 16 CONCLUSION

Cancer patients are at an exceptionally high risk of developing SARS‐CoV‐2 infection and are also likely to have higher morbidity and mortality, prompting the need for special attention to be paid to this population. Different strategies can be undertaken to manage cancer patients during the COVID‐19 pandemic. Implementing strict personal precautions for every cancer patient can be one strategy, and providing more intensive care and treatment to cancer patients infected with SARS‐CoV‐2 can be considered another useful strategy. In brief, it is recommended that curative cancer treatment should be continued, despite the potential higher risk of being infected with SARS‐CoV‐2 during anticancer therapy. Nevertheless, surgeries may be postponed based on the clinicians’ judgment, and this at‐risk population should be prioritized in the vaccination program.

## CONFLICT OF INTEREST

TTS reports that he provides strategic and scientific recommendations as a member of the Advisory Board and speaker for Novocure, Inc. and also as a member of the Advisory Board to Galera Therapeutics, which are not in any way associated with the content or disease site as presented in this manuscript. All other authors have no relevant financial interests to be declared.

## AUTHOR CONTRIBUTIONS

Zeinab Mohseni Afshar: Conceptualization, Writing—Original Draft; Rezvan Hosseinzadeh: Visualization, Writing—Review & Editing; Mohammad Barary: Investigation, Writing—Original Draft, Writing—Review & Editing; Soheil Ebrahimpour: Investigation, Writing—Original Draft; Amirmasoud Alijanpour: Writing—Review & Editing; Babak Sayad: Investigation, Writing—Original Draft; Dariush Hosseinzadeh: Visualization, Writing—Review & Editing; Seyed Rouhollah Miri: Writing—Review & Editing; Terence T. Sio: Writing—Review & Editing; Mark J. M. Sullman: Writing—Review & Editing; Kristin Carson‐Chahhoud: Writing—Review & Editing; Arefeh Babazadeh: Conceptualization, Writing—Original Draft, and Supervision.

## Data Availability

Data sharing is not applicable to this article as no new data were created or analyzed in this study.
